# Physical Activity and Different Concepts of Fall Risk Estimation in Older People–Results of the ActiFE-Ulm Study

**DOI:** 10.1371/journal.pone.0129098

**Published:** 2015-06-09

**Authors:** Jochen Klenk, Ngaire Kerse, Kilian Rapp, Thorsten Nikolaus, Clemens Becker, Dietrich Rothenbacher, Richard Peter, Michael Dieter Denkinger, the ActiFE Study Group

**Affiliations:** 1 Institute of Epidemiology and Medical Biometry, Ulm University, Ulm, Germany; 2 Department of Clinical Gerontology and Rehabilitation, Robert-Bosch-Hospital, Stuttgart, Germany; 3 Department of General Practice and Primary Health Care, School of Population Health, University of Auckland, Auckland, New Zealand; 4 Agaplesion Bethesda Clinic, Ulm, Germany; 5 Institute of the History, Philosophy and Ethics of Medicine, Ulm University, Ulm, Germany; Nathan Kline Institute and New York University School of Medicine, UNITED STATES

## Abstract

**Objectives:**

To investigate the relationship between physical activity and two measures of fall incidence in an elderly population using person-years as well as hours walked as denominators and to compare these two approaches.

**Design:**

Prospective cohort study with one-year follow-up of falls using fall calendars. Physical activity was defined as walking duration and recorded at baseline over one week using a thigh-worn uni-axial accelerometer (activPAL; PAL Technologies, Glasgow, Scotland). Average daily physical activity was extracted from these data and categorized in low (0–59 min), medium (60–119 min) and high (120 min and more) activity.

**Setting:**

The ActiFE Ulm study located in Ulm and adjacent regions in Southern Germany.

**Participants:**

1,214 community-dwelling older people (≥65 years, 56.4% men).

**Measurements:**

Negative-binomial regression models were used to calculate fall rates and incidence rate ratios for each activity category each with using (1) person-years and (2) hours walked as denominators stratified by gender, age group, fall history, and walking speed. All analyses were adjusted either for gender, age, or both.

**Results:**

No statistically significant association was seen between falls per person-year and average daily physical activity. However, when looking at falls per 100 hours walked, those who were low active sustained more falls per hours walked. The highest incidence rates of falls were seen in low-active persons with slow walking speed (0.57 (95% confidence interval (95% CI): 0.33 to 0.98) falls per 100 hours walked) or history of falls (0.60 (95% CI: 0.36 to 0.99) falls per 100 hours walked).

**Conclusion:**

Falls per hours walked is a relevant and sensitive outcome measure. It complements the concept of incidence per person years, and gives an additional perspective on falls in community-dwelling older people.

## Introduction

Falls are a major cause of injury and disability in older people and can result in serious health and social consequences such as fractures, poor quality of life, loss of independence, and nursing home admission [1,2]. Depending on the setting, fall incidence varies [1]. In community-dwelling older populations about one out of 3 persons fall each year, half of them being recurrent fallers [3,4]. Between 5 to 10% of all falls lead to fractures and more than 90% of all hip fractures result from falls [3–5].

Poor physical performance, particularly lower limb and balance problems are strong risk factors for falls [6]. Inactivity seems to be also related to an increased risk of falling probably through frailty and muscle weakness [7]. Frequent physical activity (PA) is recommended to reduce health risks like cardiovascular disease and diabetes, but also to reduce falls [8] and is a major target in the International Classification of Functioning, Disability and Health (ICF) framework to maintain or improve social participation and independent living.

There is an ongoing debate as to whether the association between physical activity and risk of falling is linear or u-shaped, that is, low activity may carry a different risk of falling compared with moderate activity and different again from those with high levels of activity. A review about the relationship between PA and risk for falls found inconsistent results [9]. Several recent studies show that those with greater levels of habitual activity sustain fewer falls or recurrent falls [10–13] whereas other studies report that those with higher levels of activity have more falls [14–17].

Physical activity in general is positively associated with physical performance and muscle strength. Therefore, increasing physical activity could reduce the number of falls. On the other hand physical activity might increase the exposure to situations associated with falls, which may in turn increase the number of falls. This was observed during a randomized control trial from Ebrahim et al. aiming to improve bone mineral density by increasing the amount of walking [18]. Unexpectedly the number of injurious falls significantly increased in the intervention group. It seems that PA and risk of falls have a complex, probably non-linear relationship and may require additional consideration on both ends of the PA distribution curve.

To date, most studies consider total time of observation to calculate fall rates within their cohorts. However, this does not accurately reflect the actual time that participants are at risk. Considering a bedridden person the risk of falling is near to zero. To shed further light on the complex relationship between PA and falls, a measure expressing risk related to exposure time may be useful. One third to one half of falls among people aged 65years and older occur while walking [3,19]. Therefore, walking duration may be a surrogate for exposure time to risk of falls. From a public health and quality of life perspective falls per hours walked might serve as another meaningful measure in addition to falls per person year.

Wijlhuizen and colleagues already addressed this problem by calculating falls per exposure time in addition to falls per person years and showed a stronger association between balance control difficulty and falls per exposure time compared to falls per person years [20]. However, they used a physical activity questionnaire to determine active days which is less accurate in terms of activity duration and known to have a potential bias (e.g. overestimation of vigorous-intensity activities and underestimation of habitual, daily activity like walking) [21]. To more accurately reflect the relationship between physical activity and falls, the actual time under risk measured by wearable sensor technology could be taken into account.

The aim of this study was to investigate the relationship between physical activity and two measures of fall incidence in a population of community-dwelling older adults using total time under observation as well as falls per time doing objectively measured physical activity and to demonstrate the different perspectives of these two approaches when assessing rate of falls.

## Materials and Methods

### Study population

The ActiFE Ulm (Activity and Function in the Elderly in Ulm) study is a population-based cohort study in older people (≥65 years), located in Ulm and adjacent regions in Southern Germany. A random-sample of 7,624 non-institutionalized inhabitants was contacted by mail and invited to participate. Exclusion criteria were: being in residential care, severe deficits in cognition or serious German language difficulties. Between March 2009 and April 2010, 1,506 eligible individuals agreed to participate and underwent baseline assessments (participation rate: 19.8%). The cohort and the measures taken were previously described [22]. In brief, baseline assessments were completed by trained research assistants using standardised methods. Age, gender, and history of falls (at least one fall during the last 12 months) were ascertained by self-report. Fear of falling was measured using the validated and commonly used Falls Efficacy Scale-International, short version (Short FES-I) [23]. Physical performance assessment consisted of the short physical performance battery for the lower extremities as well as handgrip strength (JAMAR dynamometer, Sammons Preston, Bolingbrook, Illinois) for the upper extremities [24].

All participants provided written informed consent. The ethical committee of Ulm University approved the study (application no. 318/08).

### Physical activity measurement

PA was measured using a validated uni-axial accelerometer (activPAL, PAL Technologies Ltd., Glasgow, UK) worn on the thigh [25,26]. The device was attached using waterproof adhesive tape and was not removed during sleep or bathing. Participants were instructed to wear the sensor over 24h for 7 consecutive days. Only days with activity measurement over the full 24 hours were considered as a valid day and included in the analysis. Accordingly, the first and the last day of the assessment period were excluded. The data processing algorithm detects upright posture as well as walking patterns and classified the activity into three categories: (1) lying or sitting, (2) standing and (3) walking. Average daily walking duration in hours per day was used in this study to quantify the individual’s physical activity level. Since a previous analysis showed that physical activity on Sundays was considerably different to those on other days of the week, the average physical activity within one week was calculated only for individuals with at least one measurement on a weekday and a Sunday, respectively. Overall, 5 or more complete days were available from 95% of the participants. For stratified analyses average daily physical activity was categorized in three activity groups: low (0–59 minutes), medium (60–119 minutes), and high (120 minutes and longer). Since there were no established cut-points and the results should be easy to communicate a pragmatic categorisation based on hours was selected.

### Falls

Fall rates were assessed prospectively over 12 months starting immediately after the PA measurement using weekly fall calendars [27]. Participants were asked to record the date and any fall-related injuries. Every three months the calendars were sent back to the study centre. Participants were telephoned if calendars were not returned or if information was incomplete about falls.

Fall rates were calculated in two ways: (1) falls per person-year and (2) falls per 100 hours walked. Total hours walked were estimated by multiplying the average daily physical activity as measured by the accelerometer by total number of observed days.

Participants with missing data on falls (n = 90) or who did not fulfil the minimum requirements for time of physical activity monitoring (n = 234) were excluded (in total n = 292). Compared to the study population, people excluded were more often female (45.9% vs. 42.9%), older (76.0 vs. 75.6 years), were more likely to have fallen previously (38.4% vs. 33.4%), and had lower physical performance scores: walking speed (0.95 vs. 0.98 meters per second (m/s)), handgrip strength (31.1 vs. 32.3 kg), five chair rise (11.7 vs. 11.3 second (s)). Fear of falling was comparable between those with complete data and those without.

### Statistical analysis

Adjusted incidence rates for falls as well as raw and adjusted incidence rate ratios (IRR) with 95% confidence intervals (95% CI) were calculated for falls by each category of average daily physical activity using negative-binomial regression models. Analyses were stratified for gender, three age groups (65–69 years, 70–79 years and 80–91 years), history of falls (yes or no), and walking speed ≤0.8 m/s (yes or no) as a measure of physical performance. The cut-off value for walking speed was selected based upon results of a previous publication [28]. Incidence rates and incidence rate ratios were adjusted for age (gender stratification), gender (age stratification) or both (fall history and walking speed stratification). Analyses to calculate the fall rate were performed first using person-years as the denominator to get falls per person-years; and then hours walked as a denominator yielding falls per 100 hours walked.

All analyses were performed using SAS 9.2.

## Results

The study population consisted of 693 men and 521 women (mean age = 75.6 (SD = 6.5) years) with data on physical activity and falls (Table 1). One third of the participants had at least one fall within the year prior to the baseline assessment. The average daily physical activity was 104.9 (SD = 41.0) minutes for men and 103.5 (SD = 38.9) minutes for women. More than half of the participants walked between one and two hours a day, about 13% less than one hour a day and about 30% more than two hours per day. The average walking speed was 0.98 (SD = 0.28) m/s. During 12 months follow-up 388 (31.9%) people fell at least once. The total number of falls was 706 during a total observation time of 1,164 years with an average observation time per person of 349.9 (SD = 67.6) days. Almost 90% of the participant had complete fall calendar recordings over at least 52 weeks. Further characteristics of the study population are presented in Table 1.

**Table 1:** Characteristics of study population (n = 1,214).

		Men	Women	Total
		(n = 693)	(n = 521)	(n = 1,214)
Age, mean (SD)	years	76.0 (6.40)	75.1 (6.57)	75.6 (6.49)
n (%)	65–69	147 (21.2)	137 (26.3)	284 (23.4)
	70–79	311 (44.9)	251 (48.2)	562 (46.3)
	≥80	235 (33.9)	133 (25.5)	368 (30.3)
History of falls (last 12 months), n (%)	yes	214 (30.9)	192 (36.9)	406 (33.4)
Short FES-I, median (Q1-Q3)		7 (7–8)	7 (7–9)	7 (7–8)
Habitual walking speed, mean (SD)	m/s	0.99 (0.28)	0.96 (0.29)	0.98 (0.28)
Hand grip strength, mean (SD)	kg	38.9 (9.35)	23.6 (6.46)	32.3 (11.2)
5-chair-rise, mean (SD)	s	11.1 (3.45)	11.6 (3.72)	11.3 (3.58)
Average daily physical activity, mean (SD)	min	104.9 (41.0)	103.5 (38.9)	104.3 (40.1)
n (%)	0–60 min	90 (13.0)	69 (13.2)	159 (13.1)
	60–120 min	378 (54.6)	292 (56.1)	670 (55.2)
	≥120 min	225 (32.5)	160 (30.7)	385 (31.7)
≥5 days physical activity monitoring, n (%)		656 (94.7)	498 (95.6)	1,154 (95.1)
Median observation time (Q1-Q3)	days	370 (195–370)	370 (140–370)	370 (171–370)
≥52 weeks fall calendar, n (%)		616 (88.9)	473 (90.8)	1,089 (89.7)
Average observation time per subject	days	348.5 (69.1)	351.7 (65.6)	349.9 (67.6)
Total observation time	days	241,507	183,222	424,729
Number of falls, n		381	325	706
Number of first falls, n		200	188	388

**Fig 1:**
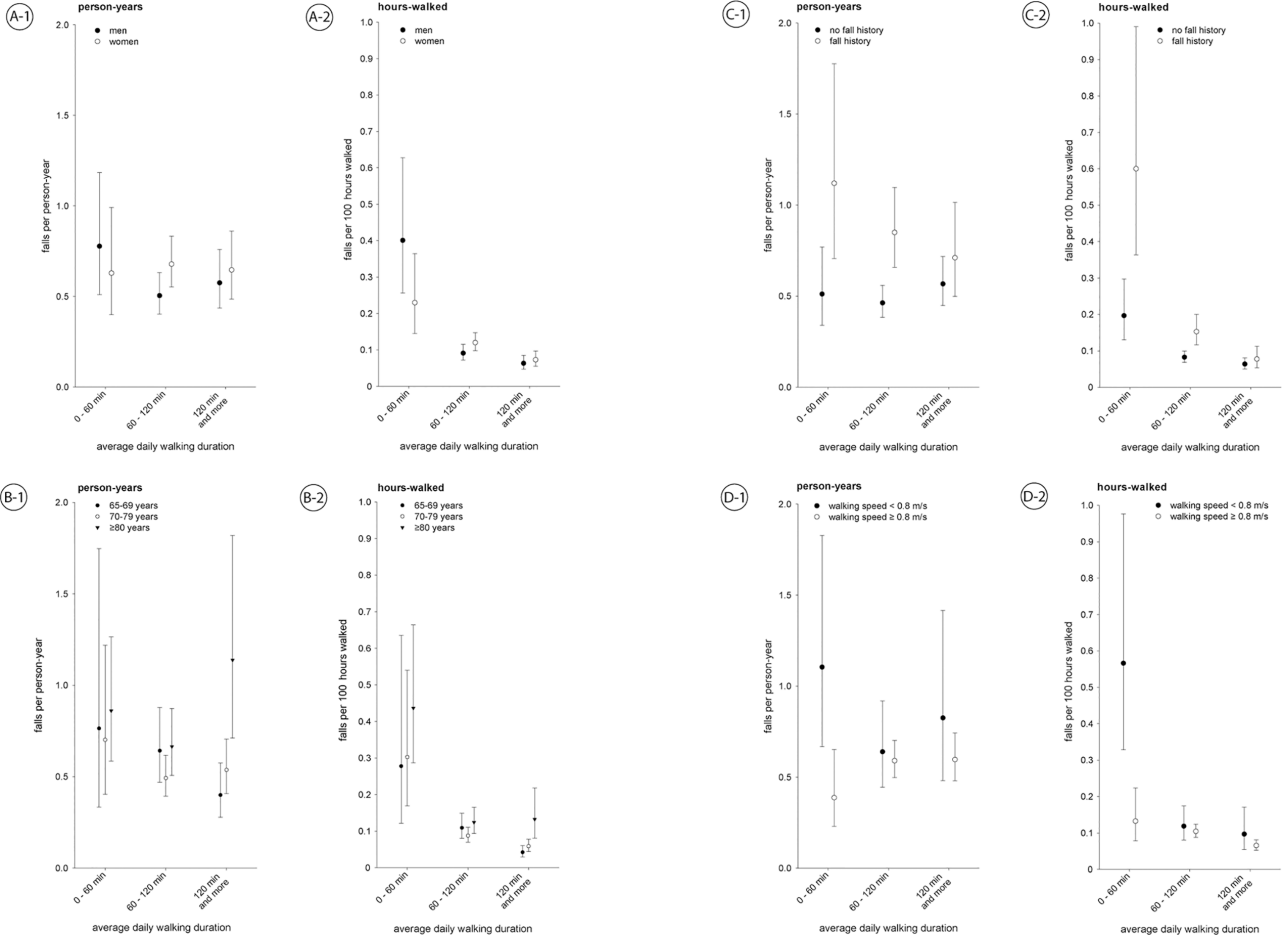
Fall rates and 95% confidence intervals for falls per person-year and falls per 100 hours walked stratified for gender, age group, fall history, and walking speed. Gender-stratified model adjusted for age, age-stratified model adjusted for gender, fall history- and walking speed-model adjusted for age and gender.

Fig 1 shows both fall rates: falls per person-year and falls per 100 hours walked for the three different ‘average daily physical activity’ groups stratified for gender, age group, fall history and walking speed <0.8 m/s.

No statistically significant association was seen between the rate of falls per person-year and average daily physical activity for all stratified analyses (Fig 1). The rate of falls per person-year for low (0–59 minutes), medium (60–119 minutes) and high active (120 minutes and more) men was 0.78 (95% CI: 0.51; 1.18), 0.50 (95% CI: 0.40; 0.63), 0.58 (95% CI: 0.44; 0.76) and in women was 0.63 (95% CI: 0.40; 0.99), 0.68 (95% CI: 0.55; 0.83), 0.65 (95% CI: 0.48; 0.86), respectively.

Considering the falls per 100 hours walked, those who were low active sustained more falls per hours walked. In men falls per 100 hours walked for low, medium and high active people was 0.40 (95% CI: 0.26; 0.63), 0.09 (95% CI: 0.07; 0.12), 0.06 (95% CI: 0.05; 0.08) and for women was 0.23 (95% CI: 0.14; 0.36), 0.12 (95% CI: 0.10; 0.14), 0.07 (95% CI: 0.05; 0.10), respectively.

Those who were low active (walked less than one hour per day) had significantly more falls per hours walked, indicated by non-overlapping confidence intervals, for men and women, and for those with previous falls and no previous falls, people aged 70 and older, and people with a walking speed <0.8 m/s. Participants with a walking speed of at least 0.8 m/s walking two hours or more per day had a significantly lower rate of falls per hours walked compared to people with a lower walking duration. High active people with a history of falls had nearly the same fall rates as medium and high active people with no fall history. Considering low active people, significantly higher fall rates were seen for people with a history of falls compared to those with no fall history as well as for people with low walking speed compared to those with high walking speed.

Table 2 shows the corresponding unadjusted and adjusted incidence rate ratios between different physical activity levels for both approaches. Considering falls per person-year only men and those with slow walking speed had a significantly higher rate of falls per person-year if they were in the low activity group. Statistical significance was lost after adjustment for age or gender. Looking at falls per hours walked, all subgroups except people whose walking speed was at least 0.8 m/s had a higher rate of falls per hours walked if they were low active compared with moderate and high active groups. Adjustment did not change the estimates considerably. Being high active was associated with a reduced risk of falling per hours walked in women, people aged 65–79 years, people who had a previous fall, and people whose walking speed was at least 0.8 m/s.

**Table 2:** Incidence rate ratios (IRR) and 95% confidence intervals (95% CI) from unadjusted and adjusted Poisson regression models using person-year and hours walked as denominator stratified for gender, age, fall history and habitual walking speed based on one year observation time.

					Unadjusted IRR (95% CI)		Adjusted IRR (95% CI)*	
	Daily physical activity [min]	No. of incident falls	Person years	Hours walked	Falls per total observation time	Falls per hours walked	Fall per total observation time	Falls per hours walked
Men	0–59	79	84.0	18494	**1.77 (1.11; 2.82)**	**5.19 (3.16; 8.52)**	1.54 (0.96; 2.46)	**4.40 (2.67; 7.23)**
	60–119	180	358.3	166135	1.00^#^	**1.00** ^#^	1.00^#^	1.00^#^
	≥120	122	219.4	162917	1.06 (0.74; 1.52)	**0.64 (0.44; 0.93)**	1.14 (0.80; 1.63)	0.69 (0.48; 1.01)
Women	0–59	40	60.2	12593	0.96 (0.59; 1.56)	**2.07 (1.27; 3.39)**	0.93 (0.56; 1.53)	**1.91 (1.15; 3.17)**
	60–119	188	286.5	124759	1.00^#^	**1.00** ^#^	1.00^#^	**1.00** ^#^
	≥120	97	155.2	101800	0.94 (0.66; 1.33)	**0.59 (0.42; 0.83)**	0.95 (0.67; 1.35)	**0.61 (0.43; 0.86)**
65–69 years	0–59	14	16.9	3803	1.18 (0.48; 2.87)	**2.51 (1.03; 6.08)**	1.19 (0.49; 2.87)	**2.54 (1.05; 6.13)**
	60–119	89	135.5	60810	1.00^#^	**1.00** ^#^	1.00^#^	**1.00** ^#^
	≥120	54	124.6	91907	**0.62 (0.38; 0.997)**	**0.39 (0.24; 0.62)**	0.62 (0.39; 0.999)	**0.39 (0.24; 0.62)**
70–79 years	0–59	29	41.6	10044	1.40 (0.77; 2.54)	**3.42 (1.84; 6.37)**	1.42 (0.78; 2.59)	**3.45 (1.85; 6.43)**
	60–119	149	308.2	142856	1.00^#^	**1.00** ^#^	1.00^#^	**1.00** ^#^
	≥120	103	193.2	136859	1.08 (0.76; 1.54)	**0.67 (0.47; 0.96)**	1.09 (0.77; 1.55)	**0.67 (0.47; 0.96)**
80–90 years	0–59	76	85.8	17240	1.30 (0.81; 2.07)	**3.53 (2.13; 5.83)**	1.29 (0.81; 2.07)	**3.51 (2.12; 5.81)**
	60–119	130	201.2	87228	1.00^#^	1.00^#^	1.00^#^	1.00^#^
	≥120	62	56.9	35951	1.72 (1.00; 2.94)	1.07 (0.61; 1.89)	1.71 (0.998; 2.94)	1.07 (0.60; 1.89)
No fall history	0–59	52	86.0	19133	1.30 (0.84; 2.01)	**2.91 (1.86; 4.54)**	1.11 (0.71; 1.73)	**2.38 (1.51; 3.75)**
	60–119	198	434.7	202676	1.00^#^	**1.00** ^#^	1.00^#^	1.00^#^
	≥120	140	262.3	186480	1.16 (0.86; 1.57)	**0.72 (0.53; 0.98)**	1.23 (0.91; 1.66)	0.77 (0.57; 1.05)
Fall history	0–59	67	58.2	11954	1.28 (0.77; 2.12)	**3.94 (2.29; 6.79)**	1.32 (0.78; 2.22)	**3.93 (2.24; 6.90)**
	60–119	170	210.1	88218	1.00^#^	**1.00** ^#^	1.00^#^	**1.00** ^#^
	≥120	79	112.3	78237	0.83 (0.54; 1.28)	**0.50 (0.32; 0.79)**	0.84 (0.54; 1.30)	**0.51 (0.32; 0.80)**
Walking speed^§^	0–59	77	66.6	12722	**1.75 (1.01; 3.04)**	**5.05 (2.78; 9.17)**	1.73 (0.99; 3.01)	**4.77 (2.62; 8.68)**
<0.8 m/s	60–119	93	157.1	68789	1.00^#^	1.00^#^	1.00^#^	1.00^#^
	≥120	43	54.1	38294	1.32 (0.71; 2.45)	0.84 (0.44; 1.62)	1.29 (0.69; 2.40)	0.82 (0.42; 1.57)
Walking speed^§^	0–59	24	61.0	15477	0.67 (0.39; 1.16)	1.32 (0.76; 2.30)	0.66 (0.38; 1.14)	1.27 (0.73; 2.20)
≥0.8 m/s	60–119	266	466.8	213272	1.00^#^	1.00^#^	1.00^#^	1.00^#^
	≥120	169	304.7	215978	0.95 (0.72; 1.25)	**0.58 (0.44; 0.77)**	1.01 (0.77; 1.33)	**0.63 (0.48; 0.82)**

* Gender-stratified model adjusted for age, age-stratified model adjusted for gender, fall history- and walking speed-stratified model adjusted for age and gender

^#^ Reference group

^§^ Missing values for walking speed (n = 58)

## Discussion

Our study did not find an association between average daily physical activity and falls per person-year. However, a clear relationship was observed between another risk measure taking exposure time, physical activity, into account, namely falls per 100 hours walked. Those who were low active (walked less than one hour per day) had significantly more falls per hours walked compared to high active individuals. The highest rate of falls per 100 hours walked was seen in low active persons with slow walking speed. This additional measure may give further insight into the complex relationship between PA and falls. It also may help to quantify the changes in falls risk related to specific activity interventions.

There are only a few studies reporting a direct measurement of physical activity in older people [29,30]. Levels of activity reported here are high compared to other cohorts with the majority of people walking more than one hour per day. Falls during the prior 12 months in the current study were experienced by 33% and the rate of falls occurring was 1.8 per person year [31]. This is comparable to other community-dwelling populations of older people [32–34].

The existing literature about physical activity and falls has used total observation time as the denominator to estimate fall risk i.e. falls per person-year or time to first fall. In community-dwelling older people three large cohort studies found an inconsistent relationship between falls and level of self-reported habitual physical activity [11,12,15]. The most active quartile in the Osteoporotic Fractures in Men Study had a significantly increased fall risk of 1.18 (95% CI: 1.07; 1.29) compared to the least active quartile [15]. In contrast, Heesch et al. found a significantly decreased Odds Ratio for falls for men and women with high levels of physical activity, 0.67 (95% CI: 0.47; 0.95) [11]. In the Longitudinal Aging Study, Amsterdam, physical activity was not significantly associated with falls but was with recurrent falls [12]. Jefferis et al. reported a positive association between objectively measured physical activity and falls per person-years in men without mobility limitation and a reverse association in persons with mobility limitations [17].

Considering falls per person-year our data suggests no association between physical activity level and falls. This is in line with findings from a randomized control trial aiming to increase physical activity in inactive community-dwelling older persons [35]. The authors did not find a difference in fall rates between intervention and control group in the follow-up period. The contrasting results observed may be due to different approaches used to assess physical activity and falls. Most previous studies have used questionnaires to measure physical activity with often retrospective methods of ascertaining falls, which may have introduced a bias [21]. A fall calendar was only used by Peeters et al. [12]. Recalling fall events over long periods, i.e. 12 months, seems to underestimate the true fall rate substantially, especially in cognitively impaired persons [27,36].

We investigated an additional concept, fall rate per hours walked. The approach is similar to that published by Wijlhuizen and colleagues but uses physical activity measured by sensor technology instead of a questionnaire to determine exposure time [20]. This concept seems more responsive and may give a new perspectives on physical activity, fall hazards and related risks. Studies show that most falls occur during transfer, turning or walking [3,19]. Falls per person-year does not account for the individual exposure to these activities and thereby does not consider an important aspect regarding burden of falls: activity duration free of falls. In the context of the ICF, activity is directly related to participation. An intervention might not decrease the number of falls per person-year but increase the hours walked till a fall happens, which might lead to a larger degree of participation and better quality of life.

The inverse of falls per hours walked is the amount of activity completed without a fall. Fall-free physical activity seems to be a more precise measure to quantify the association between various hazards and risk of falls. It might be a useful outcome measure for epidemiological studies and trials of falls prevention interventions. The utility of this parameter has to be examined in future studies and also validated against injury and hospitalization from falls in further studies. This new concept might add greater precision and useful information in addition to the traditional measures.

The measure depends upon accurate assessment of activity duration, in this case completed with an accelerometer. However, it might also be possible to use activity time measured with questionnaires to calculate falls per hours walked. This would increase the applicability especially in clinical settings. The validity of this more subjective measure will be analyzed in a future study.

### Strengths and Limitations

Major strengths of this study are the objectively measured physical activity and prospectively assessed falls over one year in a large and well-described population-based cohort. The method of fall calendars is the most accurate way to measure falls according to the PROFANE recommendations ensuring precision in data collection [27].

The fact that walking was only measured during one week and extrapolated over one year may be considered a limitation of the study. Assessment at several points throughout the year may have improved the results. However, a one-week PA measurement seems to be adequate to assess the average activity level of an individual and is in line with current recommendations [37]. Although accelerometry currently seems to be one of the most reasonable methods to quantify PA in observational studies [38], the ability to detect steps decreases at slow walking speed [26]. This may have biased the results, as underlying disease could lead to a reduction in gait speed and may also increase risk of falling meaning the increased risk is related to the disease rather than the walking speed.

In addition, knowledge of being under observation could have increased PA [39]. However, the observation period was one week for most subjects and day of measurement did not show any effects of reactivity in this cohort (data not shown). Furthermore, estimates may vary between seasons due to a lower physical activity in winter [40]. This should not have biased the results since measurements were equally distributed over the whole year. Finally, the results of our study are limited to community-dwelling older people. The relationship between falls and activity level may be different in residents of nursing homes [41].

## Conclusion

Falls per hours walked is a useful and precise outcome measure to quantify risk of falls in community-dwelling older people. It might also help to guide development of more personalized fall prevention programs. In our study physical activity was not a risk factor for falls per person-year, but was for falls per hours walked. The highest rates of falls were observed in low active older persons with slow walking speed or a history of falls. These findings suggest that prevention programs aiming to increase physical activity in this group might have to incorporate additional components like progressive balance training. Fall-free walking time seems to be a new additional outcome measure for intervention studies and an important attribute to indicate participation in life for older people.

## References

[1] Rubenstein LZ. Falls in older people: epidemiology, risk factors and strategies for prevention. Age Ageing. 2006;35 Suppl 2: ii37–ii41. 10.1093/ageing/afl084 16926202

[2] Jørstad EC, Hauer K, Becker C, Lamb SE. Measuring the psychological outcomes of falling: a systematic review. J Am Geriatr Soc. 2005;53: 501–510. 10.1111/j.1532-5415.2005.53172.x 15743297

[3] Tinetti ME, Speechley M, Ginter SF. Risk factors for falls among elderly persons living in the community. N Engl J Med. 1988;319: 1701–1707. 10.1056/NEJM198812293192604 3205267

[4] Campbell AJ, Borrie MJ, Spears GF, Jackson SL, Brown JS, Fitzgerald JL. Circumstances and consequences of falls experienced by a community population 70 years and over during a prospective study. Age Ageing. 1990;19: 136–141. 2337010 10.1093/ageing/19.2.136

[5] Parkkari J, Kannus P, Palvanen M, Natri A, Vainio J, Aho H, et al Majority of hip fractures occur as a result of a fall and impact on the greater trochanter of the femur: a prospective controlled hip fracture study with 206 consecutive patients. Calcif Tissue Int. 1999;65: 183–187. 10441647 10.1007/s002239900679

[6] Deandrea S, Lucenteforte E, Bravi F, Foschi R, La Vecchia C, Negri E. Risk factors for falls in community-dwelling older people: a systematic review and meta-analysis. Epidemiology. 2010;21: 658–668. 10.1097/EDE.0b013e3181e89905 20585256

[7] Campbell AJ, Buchner DM. Unstable disability and the fluctuations of frailty. Age Ageing. 1997;26: 315–318. 9271296 10.1093/ageing/26.4.315

[8] Khan KM, Liu-Ambrose T, Donaldson MG, McKay HA. Physical activity to prevent falls in older people: time to intervene in high risk groups using falls as an outcome. Br J Sports Med. 2001;35: 144–145. 11375870 10.1136/bjsm.35.3.144 PMC1724338

[9] Gregg EW, Pereira MA, Caspersen CJ. Physical activity, falls, and fractures among older adults: a review of the epidemiologic evidence. J Am Geriatr Soc. 2000;48: 883–893. 10968291 10.1111/j.1532-5415.2000.tb06884.x

[10] Graafmans WC, Lips P, Wijlhuizen GJ, Pluijm SM, Bouter LM. Daily physical activity and the use of a walking aid in relation to falls in elderly people in a residential care setting. Z Gerontol Geriatr. 2003;36: 23–28. 10.1007/s00391-003-0143-8 12616404

[11] Heesch KC, Byles JE, Brown WJ. Prospective association between physical activity and falls in community-dwelling older women. J Epidemiol Community Health. 2008;62: 421–426. 10.1136/jech.2007.064147 18413455

[12] Peeters GMEE, van Schoor NM, Pluijm SMF, Deeg DJH, Lips P. Is there a U-shaped association between physical activity and falling in older persons? Osteoporos Int. 2010;21: 1189–1195. 10.1007/s00198-009-1053-4 19756832 PMC2906720

[13] Mertz KJ, Lee D-C, Sui X, Powell KE, Blair SN. Falls among adults: the association of cardiorespiratory fitness and physical activity with walking-related falls. Am J Prev Med. 2010;39: 15–24. 10.1016/j.amepre.2010.03.013 20547276 PMC2897244

[14] Li W, Keegan THM, Sternfeld B, Sidney S, Quesenberry CP Jr, Kelsey JL. Outdoor falls among middle-aged and older adults: a neglected public health problem. Am J Public Health. 2006;96: 1192–1200. 10.2105/AJPH.2005.083055 16735616 PMC1483851

[15] Chan BKS, Marshall LM, Winters KM, Faulkner KA, Schwartz AV, Orwoll ES. Incident fall risk and physical activity and physical performance among older men: the Osteoporotic Fractures in Men Study. Am J Epidemiol. 2007;165: 696–703. 10.1093/aje/kwk050 17194749

[16] Lawton BA, Rose SB, Elley CR, Dowell AC, Fenton A, Moyes SA. Exercise on prescription for women aged 40–74 recruited through primary care: two year randomised controlled trial. BMJ. 2008;337: a2509 10.1136/bmj.a2509 19074218 PMC2769033

[17] Jefferis BJ, Merom D, Sartini C, Wannamethee SG, Ash S, Lennon LT, et al Physical Activity and Falls in Older Men: The Critical Role of Mobility Limitations. Med Sci Sports Exerc. 2015; 10.1249/MSS.0000000000000635 PMC5131688 25668406

[18] Ebrahim S, Thompson PW, Baskaran V, Evans K. Randomized placebo-controlled trial of brisk walking in the prevention of postmenopausal osteoporosis. Age Ageing. 1997;26: 253–260. 10.1093/ageing/26.4.253 9271287

[19] Berg WP, Alessio HM, Mills EM, Tong C. Circumstances and consequences of falls in independent community-dwelling older adults. Age Ageing. 1997;26: 261–268. 9271288 10.1093/ageing/26.4.261

[20] Wijlhuizen GJ, Chorus AMJ, Hopman-Rock M. The FARE: A new way to express FAlls Risk among older persons including physical activity as a measure of Exposure. Preventive Medicine. 2010;50: 143–147. 10.1016/j.ypmed.2009.12.014 20045023

[21] Ainsworth BE. How do I measure physical activity in my patients? Questionnaires and objective methods. Br J Sports Med. 2009;43: 6–9. 10.1136/bjsm.2008.052449 18718977

[22] Denkinger MD, Franke S, Rapp K, Weinmayr G, Duran-Tauleria E, Nikolaus T, et al Accelerometer-based physical activity in a large observational cohort—study protocol and design of the activity and function of the elderly in Ulm (ActiFE Ulm) study. BMC Geriatr. 2010;10: 50 10.1186/1471-2318-10-50 20663209 PMC2919539

[23] Kempen GIJM, Yardley L, van Haastregt JCM, Zijlstra GAR, Beyer N, Hauer K, et al The Short FES-I: a shortened version of the falls efficacy scale-international to assess fear of falling. Age Ageing. 2008;37: 45–50. 10.1093/ageing/afm157 18032400

[24] Guralnik JM, Simonsick EM, Ferrucci L, Glynn RJ, Berkman LF, Blazer DG, et al A short physical performance battery assessing lower extremity function: association with self-reported disability and prediction of mortality and nursing home admission. J Gerontol. 1994;49: M85–94. 8126356 10.1093/geronj/49.2.m85

[25] Grant PM, Ryan CG, Tigbe WW, Granat MH. The validation of a novel activity monitor in the measurement of posture and motion during everyday activities. Br J Sports Med. 2006;40: 992–997. 10.1136/bjsm.2006.030262 16980531 PMC2577473

[26] Ryan CG, Grant PM, Tigbe WW, Granat MH. The validity and reliability of a novel activity monitor as a measure of walking. Br J Sports Med. 2006;40: 779–784. 10.1136/bjsm.2006.027276 16825270 PMC2564393

[27] Lamb SE, Jørstad-Stein EC, Hauer K, Becker C. Development of a common outcome data set for fall injury prevention trials: the Prevention of Falls Network Europe consensus. J Am Geriatr Soc. 2005;53: 1618–1622. 10.1111/j.1532-5415.2005.53455.x 16137297

[28] Rapp K, Klenk J, Benzinger P, Franke S, Denkinger MD, Peter R, et al Physical performance and daily walking duration: associations in 1271 women and men aged 65–90 years. Aging Clin Exp Res. 2012;24: 455–460. 10.3275/8264 22313581

[29] Taraldsen K, Chastin SFM, Riphagen II, Vereijken B, Helbostad JL. Physical activity monitoring by use of accelerometer-based body-worn sensors in older adults: a systematic literature review of current knowledge and applications. Maturitas. 2012;71: 13–19. 10.1016/j.maturitas.2011.11.003 22134002

[30] Kerse N, Elley CR, Robinson E, Arroll B. Is physical activity counseling effective for older people? A cluster randomized, controlled trial in primary care. J Am Geriatr Soc. 2005;53: 1951–1956. 10.1111/j.1532-5415.2005.00466.x 16274377

[31] Rapp K, Freiberger E, Todd C, Klenk J, Becker C, Denkinger M, et al Fall incidence in Germany: results of two population-based studies, and comparison of retrospective and prospective falls data collection methods. BMC Geriatr. 2014;14: 105 10.1186/1471-2318-14-10532 25241278 PMC4179843

[32] Blake AJ, Morgan K, Bendall MJ, Dallosso H, Ebrahim SB, Arie TH, et al Falls by elderly people at home: prevalence and associated factors. Age Ageing. 1988;17: 365–372. 3266440 10.1093/ageing/17.6.365

[33] Campbell AJ, Diep C, Reinken J, McCosh L. Factors predicting mortality in a total population sample of the elderly. J Epidemiol Community Health. 1985;39: 337–342. 4086965 10.1136/jech.39.4.337 PMC1052468

[34] Gillespie LD, Robertson MC, Gillespie WJ, Sherrington C, Gates S, Clemson LM, et al Interventions for preventing falls in older people living in the community. Cochrane Database Syst Rev. 2012;9: CD007146 10.1002/14651858.CD007146.pub3 22972103 PMC8095069

[35] Voukelatos A, Merom D, Sherrington C, Rissel C, Cumming RG, Lord SR. The impact of a home-based walking programme on falls in older people: the Easy Steps randomised controlled trial. Age Ageing. 2015; afu186 10.1093/ageing/afu186 25572426

[36] Zieschang T, Schwenk M, Becker C, Oster P, Hauer K. Feasibility and accuracy of fall reports in persons with dementia: a prospective observational study. Int Psychogeriatr. 2011; 1–12. 10.1017/S1041610211002122 22142666

[37] Ward DS, Evenson KR, Vaughn A, Rodgers AB, Troiano RP. Accelerometer use in physical activity: best practices and research recommendations. Med Sci Sports Exerc. 2005;37: S582–588. 16294121 10.1249/01.mss.0000185292.71933.91

[38] Janz KF. Physical activity in epidemiology: moving from questionnaire to objective measurement. Br J Sports Med. 2006;40: 191–192. 10.1136/bjsm.2005.023036 16505072 PMC2492010

[39] Clemes SA, Matchett N, Wane SL. Reactivity: an issue for short-term pedometer studies? Br J Sports Med. 2008;42: 68–70. 10.1136/bjsm.2007.038521 18178685

[40] Klenk J, Büchele G, Rapp K, Franke S, Peter R. Walking on sunshine: effect of weather conditions on physical activity in older people. J Epidemiol Community Health. 2012;66: 474–476. 10.1136/jech.2010.128090 21325149

[41] Robinovitch SN, Feldman F, Yang Y, Schonnop R, Leung PM, Sarraf T, et al Video capture of the circumstances of falls in elderly people residing in long-term care: an observational study. Lancet. 2013;381: 47–54. 10.1016/S0140-6736(12)61263-X 23083889 PMC3540102

